# A web-based workplace exercise intervention among office workers with spinal pain: Protocol of a mixed methods study

**DOI:** 10.1371/journal.pone.0325376

**Published:** 2025-06-09

**Authors:** Carlos Tersa-Miralles, Cristina Bravo, Filip Bellon, Maria Masbernat-Almenara, Francesc Rubí-Carnacea, Esther Rubinat Arnaldo

**Affiliations:** 1 Consolidated Research Group: Society, Health, Education and Culture(GESEC), Universitat de Lleida, Lleida, Spain; 2 Department of Nursing and Physiotherapy, Universitat de Lleida, Lleida, Spain; 3 Health Care Research Group Salut (GRECS), IRBLleida, Institut de Recerca Biomédica Fundació Dr. Pifarré, Lleida, Spain; 4 Center for Biomedical Research on Diabetes and Associated Metabolic Diseases (CIBERDEM), Instituto de Salud Carlos III, Barcelona, Spain; Uttara Adhunik Medical College, BANGLADESH

## Abstract

**Introduction:**

Musculoskeletal disorders are a major cause of disability worldwide, significantly impacting office workers due to prolonged sitting and lack of movement. Implementing therapeutic exercise interventions in the workplace has been identified as a feasible and cost-effective strategy to address spinal pain. However, understanding workers’ perspectives and workplace barriers is essential for designing effective interventions. This study aims to develop and evaluate a web-based workplace intervention with active breaks to reduce spinal pain among office workers.

**Methods:**

This study follows a sequential exploratory mixed-methods design. The qualitative phase will use semi-structured interviews with office workers to explore their experiences with spinal pain, active breaks, and perceived barriers to implementation. These findings will inform the development of a six-week web-based therapeutic exercise intervention, which will be evaluated through a two-arm cluster randomised controlled trial. The trial will compare an intervention group performing structured active breaks during work hours with a control group maintaining their usual routine. Primary outcomes include pain intensity (Visual Analogue Scale), spinal dysfunction (Spine Functional Index), and adherence to the program. Secondary outcomes include quality of life (EQ-5D-5L) and exercise motivation (Behavioural Regulation in Exercise Questionnaire-2). Statistical analyses will compare within- and between-group differences to assess the intervention’s effectiveness.

**Discussion:**

Web-based interventions can enhance adherence to active breaks and provide an accessible, cost-effective solution for spinal pain management in sedentary workplaces. By adopting a mixed-methods approach, this study will generate valuable insights into implementing workplace exercise interventions, taking into account workers’ expectations, workplace context, and adherence factors. Findings may inform future interventions aimed at managing musculoskeletal disorders in office workers.

**Trial registration:**

ClinicalTrials.gov NCT05571124

## Introduction

### Background

Musculoskeletal disorders are a leading cause of disability worldwide, significantly contributing to work-related disability, absenteeism, and presenteeism [1]. In Europe, musculoskeletal conditions in the workforce account for up to 2% of the Gross Domestic Product, [2] with spinal pain being the most common work-related health problem, affecting 43% of the working population [3]. Notably, up to 90% of the cases are classified as non-specific, meaning they cannot be linked to a particular pathology [4,5].

Workers who spend most of their time in front of the computer are predisposed to musculoskeletal pain due to the sedentary nature of their jobs, which involve repetitive movements, prolonged postures, and low physical activity [6]. In developed countries, more than 60% of office workers report physical problems related to musculoskeletal pain, which varies depending on their job type and screen time [7].

Several risk factors for musculoskeletal disorders have been described, such as job stress, the work environment, and job-related factors [8,9]. Biopsychosocial aspects play an essential role in physical health and the suffering of spinal pain, which includes both back and neck pain, among office workers [10,11]. Given the sedentary nature of office work, both regions are commonly affected, and addressing spinal pain comprehensively ensures that the intervention is relevant to a wider range of musculoskeletal conditions. Furthermore, factors like job dissatisfaction and inadequate social support can significantly impact the quality of life for workers dealing with chronic pain [12].

Implementing ergonomic, educational and organisational strategies can benefit the office worker in reducing musculoskeletal problems [13]. While ergonomic interventions are cost-effective and feasible, a comprehensive approach that combines multiple interventions, such as workplace exercise or physical activity programs, may be necessary to achieve meaningful reductions in musculoskeletal pain [14], a comprehensive approach that includes multiple interventions may be necessary to effectively reduce musculoskeletal problems in office workers such as workplace exercise or physical activity interventions [15,16].

Therapeutic exercise in the workplace has reported improvements in pain reduction, although there is significant heterogeneity in its implementation [17]. A lack of understanding about pain and kinesiophobia, often leads workers to remain sedentary for extended periods [18]. Also, in the CUPID study [19], it was observed that workers’ expectations and low mood can influence the disability caused by musculoskeletal pain.

To develop more effective strategies, exercise interventions should not only focus on pain reduction but also address the psychosocial factors associated with musculoskeletal disorders. These interventions can enhance workers’ self-confidence in managing their condition and promoting a proactive approach to treatment [20].

Different randomised controlled trials [21,22] demonstrated that using online tools for patients with chronic pain is an effective and low-cost alternative to conventional physiotherapy treatment in pain management and reduction. These studies used multimodal approaches, incorporating both exercise and education as core components.

Although more studies are needed to know which are the best health interventions for the workplace [23], taking into account patients’ preferences for interventions and self-management skills may be a good option to maintain high levels of treatment adherence and reduce presenteeism. Maintaining high adherence to treatment is essential for successful treatment to reduce musculoskeletal disorders [24,25].

Involving participants in the design and implementation of interventions may enhance adherence by allowing modifications to lifestyle and behaviour-related risk factors [26]. Furthermore, understanding workers’ expectations, preferences and context concerning pain and exercise would enhance motivation and adherence to treatment [27].

To further support these motivational processes, this study is informed by Self-Determination Theory (SDT) [28], which identifies three essential psychological needs: autonomy, competence, and relatedness as key drivers of sustained behavioural engagement [29]. Exploring these needs of the participants can help design more effective and personalised workplace interventions.

### Objectives

This mixed-methods study aims to develop and evaluate a web-based workplace exercise intervention designed to reduce chronic non-specific spinal pain among office workers. The study first seeks to understand the perspectives of office workers on spinal pain and active breaks, and based on these findings, the effectiveness of the intervention will be assessed in improving pain management and addressing both the physical and psychosocial aspects of musculoskeletal disorders.

## Materials and methods

This study intends to utilise a sequential exploratory mixed-methods research design [30]. Initially, the investigators will conduct a qualitative phase to explore office workers’ perceptions of spinal pain and active breaks. The findings from this phase will then inform the development of the intervention, which will be tested in a subsequent quantitative randomised controlled trial.

The study will follow a three-step method:

- Qualitative Phase: Gathering and analysing qualitative data.
- Connection Phase: Drawing from the qualitative insights, the structure and content of the web-based user-centred program.
- Quantitative Phase: The web-based program will undergo evaluation with a sample of participants followed by the collection and analysis of quantitative data.

The sequential exploratory approach ensures that the intervention is tailored to the specific needs and contexts of office workers, enhancing feasibility and adherence. This methodological framework has been widely used in public health research to integrate qualitative insights with robust quantitative evaluation [30].

### Qualitative phase

#### Design.

A qualitative study using semi-interviews will be conducted with office workers with chronic non-specific spinal pain. This phase follows a constructivist paradigm and employs a hermeneutic-interpretative phenomenological study approach to explore participants’ lived experiences. This method provides an in-depth understanding of the experiences and meanings that guide participants’ behaviour. This methodology makes it possible to capture principles, ideas and meanings from personal stories, which can be contrasted and compared with those of other participants, exposing coincidences that make it possible to create general concepts about the problem under study [31].

The qualitative phase is essential for identifying psychosocial and contextual factors influencing spinal pain among office workers, ensuring the intervention aligns with workplace realities and behavioural tendencies. Chronic pain is shaped by an interplay of biological, psychological, and social factors, affecting pain perception, adherence, and disability [32].

Following the biopsychosocial model, this phase will explore:

- Beliefs about spinal pain and its management (e.g., inevitability vs. modifiability).
- Workplace barriers and facilitators to active breaks (e.g., time constraints, ergonomics).
- Behavioural factors affecting adherence (e.g., motivation, movement-related fear).
- Expectations for the intervention and its feasibility in daily routines.

The interview guide also draws on SDT, focusing on how autonomy, competence, and relatedness influence participants’ motivation to engage in workplace exercise [29].

These insights will ensure the intervention targets pain reduction and addresses psychosocial barriers. The study will follow the SRQR checklist for rigorous qualitative reporting [33].

#### Sampling, sample size and method of approach.

The sample will be accessible from the different faculties of the University of Lleida, which will be contacted through the different managers on each campus. One manager from each campus will be contacted to facilitate communication with the research team. The principal investigator will oversee communicating with the different contacts at each centre. Semi-structured interviews will be conducted during working hours, either in the participants’ office or in an available room within the same faculty. Interviews will be conducted in a confidential setting without the presence of other university staff members.

The inclusion criteria consist of office workers from various campuses of the University of Lleida who have been experiencing spinal pain for more than three months, spend at least 80% of their working hours in a seated position, and lead a sedentary lifestyle that does not meet the physical activity guidelines proposed by the World Health Organisation (WHO). These criteria will be assessed using a recruitment form, including questions from the International Physical Activity Questionnaire (iPAQ) [34], which evaluates weekly levels of physical activity and daily step counts. Participants must engage fewer than 75 minutes of vigorous or 150 minutes of moderate activity per week and take fewer than 10,000 steps per day [35].

Exclusion criteria include individuals with rheumatologic conditions (e.g., rheumatoid arthritis, ankylosing spondylitis), central sensitization syndromes (e.g., fibromyalgia), or other chronic pain disorders requiring ongoing pharmacological treatment (e.g., opioids, muscle relaxants). Participants currently undergoing physical therapy for musculoskeletal conditions, those absent from work, or individuals unable to perform the prescribed exercises due to medical conditions safely will also be excluded. While this study addresses both low back and neck pain under the broader category of spinal pain, this decision reflects the high prevalence of pain in both regions among office workers, as prolonged sitting and poor ergonomics affect the entire spine rather than isolated segments.

The study sample will be selected using non-probabilistic convenience sampling. The final sample size will depend on data saturation, following the approach recommended by Vasileiou et al [36], in their study.

#### Data collection and analysis.

The principal investigator CTM together with CB, both experienced in qualitative research, will conduct the semi-structured interviews at each university campus to collect subjective opinions and explore different points of view of the interviewed participants, being this technique a way of obtaining valuable information for the study [37]. The interviews will be video and audio-recorded to be later systematically transcribed verbatim. Atlas.ti software will be used for coding and data management [38].

The systematic text condensation proposed by Malterud [39], based on Georgi’s psychological phenomenological method [40] will be used for the qualitative data analysis. This analysis was created to develop the information reported by the participants in the interviews taking into account the recognition and importance of the researcher’s point of view on the subject and consists of 4 steps: 1. A general impression. 2. The identification of meaning units related to the phenomenological focus studied. 3. Coding the meaning units and grouping them by themes, considering the interviews. 4. Synthesis of the themes and categories.

The interviews will explore perceptions of spinal pain, including its causes, intensity, frequency, impact on work, and management strategies. Additionally, participants will discuss posture and ergonomics, focusing on the relationship between prolonged sitting and spinal pain, workplace ergonomics, and adaptive postural changes. Another key theme will be exercise and active breaks, exploring views on exercise for pain prevention, previous experiences with active breaks, types of exercises preferred, and barriers to implementation. Finally, participants will reflect on the feasibility of a web-based intervention and the digital platform, including preferences for accessing exercise tutorials, the potential for integrating an online program into their work routine, and the perceived usefulness of digital health resources. These insights will directly inform the exercise program’s design in the quantitative phase, ensuring it aligns with participants’ needs and workplace constraints. The semi-structured interview guide is provided in S1 File.

### Connection phase

In the context of addressing musculoskeletal spinal pain among office workers engaged in sedentary work, the qualitative phase is crucial for understanding how to integrate active breaks into their daily routines effectively and ensure high adherence tailored to their specific needs.

Upon analysing the qualitative data, the researchers will develop the content and materials (such as videos and informational dossiers) for the active break intervention. These materials will be hosted on the university’s virtual campus, which participants regularly access for work-related activities. Ensuring that the materials align with the experiences and needs of office workers with spinal pain will enhance usability and engagement. The intervention’s content, delivery, and communication will be guided by themes from the qualitative analysis, especially participants’ motivational barriers, preferences, and perceived feasible exercises. For example, if time constraints are a key barrier, short, flexible sessions will be prioritised; if visual instruction is preferred, video content will be emphasised.

The intervention will be delivered via Sakai [41], an open-source Learning Management System chosen for its familiarity and ease of use. This platform was chosen to ensure familiarity and ease of use for participants, as it is already utilised in their daily work routines. The intervention will be implemented asynchronously through the Sakai platform, allowing participants to complete the exercises at flexible times during work hours. The intervention will include media content (depending on participants’ needs) featuring low-dosage exercises focused on stretching and postural strengthening. These exercises will primarily target the different spine regions and adjacent areas such as the shoulders and hips. The primary goal is to interrupt sedentary time and promote movement variability of the spine [42].

A specialised physiotherapist with expertise in occupational health will oversee the program, providing remote supervision to guide participants and address any concerns. While the intervention is self-directed, participants will have direct access to the physiotherapist via email and platform messaging for any inquiries related to exercise execution, discomfort, or adherence difficulties. Additionally, if participants experience persistent difficulties or require further support, video consultations can be arranged upon request.

The asynchronous format was chosen to maximise flexibility and accessibility, allowing participants to engage with the intervention at convenient times without requiring real-time coordination. This is particularly relevant in office settings with varying workloads, where synchronous formats could limit participation [43]. Asynchronous telehealth interventions, particularly those with well-designed, interactive platforms that provide tailored information and self-management strategies, enhance adherence and engagement by empowering participants to manage their conditions effectively. This approach maximises accessibility and minimises barriers such as irrelevant content, information overload, and the impersonal nature of telehealth. Addressing these challenges, including support for individuals with low digital literacy, is crucial to fostering consistent engagement and self-efficacy [44].

Based on the qualitative findings, strategies such as periodic reminders, motivational messages, or personalised follow-up may be used to support engagement. These approaches are supported by existing literature, which highlights their effectiveness in improving adherence in digital health interventions [45].

### Quantitative phase

#### Design.

A two-arm cluster randomised controlled trial will be conducted following the recommendations of the CONSORT checklist [46]. A six-week online intervention will be carried out using exercise videos, taking into account the current scientific evidence and the results of the previous phase. The intervention will align with established guidelines [47], which emphasises therapeutic exercise, including strengthening exercises, mobility and stretching routines, and general physical activity, to improve musculoskeletal function and address musculoskeletal disorders.

In this study aimed at reducing musculoskeletal spinal pain among university office workers, two distinct interventions are being employed: the Intervention Group and the Control Group.

The Intervention Group will implement a six-week therapeutic exercise intervention in their workplace environment, following the program content developed in the previous phase.

Conversely, participants in the Control Group will be placed on a waiting list, advised to maintain their regular daily activities, and working hours. Upon completion of the questionnaires after the six weeks, they will gain access to the platform, receiving identical content to that of the Intervention Group.

#### Recruitment and sampling method.

The recruitment process will commence after the completion of the connection phase, once the qualitative data have been analysed and integrated into the design and development of the web-based intervention platform. This ensures that participants’ perspectives and workplace realities inform the intervention before implementation.

Participants in the quantitative phase will be recruited separately from the qualitative phase, following the sequential exploratory mixed-methods design [30]. However, office workers who participated in the qualitative phase will be encouraged to join the intervention trial, allowing for continuity while maintaining the opportunity to recruit a broader sample.

The inclusion and exclusion criteria for the quantitative phase will remain identical to those of the qualitative phase to ensure consistency in participant characteristics. Recruitment will involve emailing all office workers across the University of Lleida campuses. The lead author will oversee participant selection, conducting interviews to verify eligibility based on the established criteria. During these interviews, participants will be provided with a detailed explanation of the intervention, including its purpose, procedures, and expected level of participation.

To ensure ethical compliance, all participants will be required to sign an informed consent form before enrolment in the study. The informed consent document is provided in S2 File.

#### Sample size calculation.

The GRANMO calculator has been used to obtain the sample size. The study by Kenneth et al [48], has been taken for reference with a minimum significant difference in pain intensity of 1.5 and a standard deviation of 1.5, considering an alpha risk of 0.05 and a statistical power of 95% to reject the null hypothesis, the minimum number of participants per group necessary will be 33 accounting for an expected 20% dropout rate.

#### Randomisation: type and sequence generation.

Participants in the study will be assigned to either the Intervention Group or the Control Group through cluster randomisation. The six campuses of the University will be randomised into either group to prevent contamination between groups. A 1:1 ratio will be maintained to ensure an equal distribution of campuses between the experimental and control clusters. The randomisation will be conducted using Study Randomizer, a web-based service, to ensure unbiased allocation [49].

#### Allocation concealment mechanism and implementation.

To ensure that allocation is concealed, one of the researchers will randomly create the codes in opaque envelopes with the username and password for participants to log into the web platform.

#### Blinding.

The evaluators will be blinded to the data collection as each participant will be assigned a code for identification. The participants will not be blinded as they are aware of the intervention they are carrying out.

#### Data collection and analysis.

Participants in both groups will complete self-administered online questionnaires at baseline and six weeks post-intervention.

Email reminders will be sent at each data collection time point, and if participants fail to respond, additional follow-ups will be conducted via email and, if necessary, by phone at their workplace. Upon completion, data will be securely stored, accessible only to the appointed statistician, and later exported to STATA v.16 software for analysis [50].

#### Primary outcome.

Pain intensity: Spinal pain will be assessed using the Visual Analogue Scale (VAS) [51,52], a subjective scale ranging from 0 to 10, with 0 representing “no pain” and 10 representing “the worst possible pain.” A clinically important difference (MCID) for the VAS is defined as a 30% reduction from baseline [53], which is considered a meaningful improvement in pain intensity for musculoskeletal conditions. Therefore, any reduction of 30% or greater in VAS scores will be considered a clinically significant improvement.

#### Secondary outcomes.

Spinal pain dysfunction: Spinal pain dysfunction will be assessed using the Spanish version of the Spine Functional Index (SFI) [54,55], a self-administered questionnaire that evaluates the impact of spinal conditions on daily activities and functional abilities. In the absence of an established MCID, we will apply the distribution-based approach, calculating the MCID as 0.5 times the standard deviation of baseline scores. This method is widely used in clinical research to estimate clinically meaningful changes based on the variability within the sample when anchor-based MCIDs are unavailable [56].

#### Quality of life.

Quality of life will be evaluated using the Spanish version of the EQ-5D-5L questionnaire [57,58], which covers five dimensions: mobility, self-care, daily activities, pain/discomfort, and anxiety/depression. Participants respond to the EQ-5D-5L questionnaire by indicating their level of impairment or difficulty in each dimension on a five-level scale, ranging from no problems to extreme problems. The responses provide quantifiable data that can be analysed to assess overall health status and quality of life, guiding healthcare interventions and treatment planning. Since there is no established MCID for the EQ-5D-5L in this population, we will also apply the distribution-based approach, using 0.5 times the standard deviation of baseline scores to estimate the MCID [56].

#### Exercise motivation.

Exercise motivation will be assessed using the Spanish version of the Behavioural Regulation in Exercise Questionnaire-2 (BREQ-2) [59–61], which evaluates five distinct types of motivation: intrinsic motivation, identified regulation, introjected regulation, external regulation, and amotivation. The BREQ-2 has been validated for use in individuals with chronic musculoskeletal pain, making it suitable for assessing exercise motivation in this population. The same distribution-based approach used for other measures lacking anchor-based MCIDs (i.e., 0.5 times the standard deviation of baseline scores) will be employed to determine clinically meaningful changes [56]. Significant increases in intrinsic motivation (exercising for enjoyment) and identified regulation (exercising because it is personally important) will be considered meaningful for predicting better adherence to the exercise intervention [28].

#### Adherence to the program.

Therapeutic adherence will be assessed using a self-administered diary available on the Sakai platform, where participants will record the active breaks they performed at the end of each workday. To promote adherence, reminder emails will be sent at the start and end of each week to prompt participants to complete their diary entries. Adherence data will be reviewed weekly by the research team to monitor participant engagement. If adherence remains unreported at the end of the week, a personalised follow-up email from the supervising physiotherapist will be sent at the start of the following week.

This diary serves as a tool to monitor and evaluate participants’ compliance with the intervention, providing valuable insights into their engagement with the prescribed active break regimen. The 80% adherence rate is considered satisfactory in pain management interventions [62,63].

#### Statistical analysis.

Descriptive statistics will be used to summarise the demographic and baseline characteristics of participants. Categorical variables (e.g., gender, educational level) will be compared between control and intervention group using Chi-square tests or Fisher’s exact tests, as appropriate, while continuous variables (e.g., age) will be compared using independent-samples t-tests for normally distributed variables or Mann–Whitney U tests if the data are not normally distributed. The Shapiro-Wilk test and Q-Q plots will be employed to assess normality. If the data meet normality assumptions (p > 0.05), parametric tests will be applied; otherwise, non-parametric tests will be used.

Within-group comparisons (before- vs. after-intervention) will be conducted using paired t-tests for normally distributed variables, and the Wilcoxon signed-rank test will be applied for variables not meeting normality assumptions. Changes from baseline between the intervention and control groups (before and after differences) will be evaluated using independent-samples t-tests for normally distributed data, or Mann–Whitney U tests for non-normal data. Clinically significant changes will be assessed using criteria established in previously reviewed studies.

Mixed-effects models will be employed to evaluate the effect of the intervention over time, taking into account repeated measures. A linear mixed-effects model will be applied to outcomes that are approximately normally distributed, with random intercepts included to account for within-subject variability. If an outcome clearly violates normality assumptions even after exploration or transformation, a generalized linear mixed model (GLMM) with an appropriate link function (e.g., logit for binary outcomes) may be considered. These models will adjust for potential confounders such as age, gender, educational level, spinal pathologies, job tasks, and adherence. Statistical significance will be set at a p-value of less than 0.05. Mixed-effects models are appropriate for this cluster-randomised design with repeated measures, as they account for both fixed effects and within-subject variability. Sample size was estimated assuming a moderate effect size, an ICC of 0.05, alpha = 0.05, and 80% power.

An intention-to-treat analysis will be conducted, ensuring that all participants are analysed in their originally assigned groups. Missing data will be addressed using complete-case analysis, assuming data are missing at random. Sensitivity analyses will be considered to assess the potential impact of missingness on the results. All statistical analyses will be performed using STATA v.16 software [50].

#### Ethical considerations.

This study will adhere to the principles outlined in the Declaration of Helsinki and the guidelines of Good Clinical Practice. The study protocol has obtained approval from the Research Ethics Committee (CEIm) of the Hospital Universitari Arnau de Vilanova under the reference CEIC-2842.

#### Validity and reliability/rigour.

**Fig 1:**
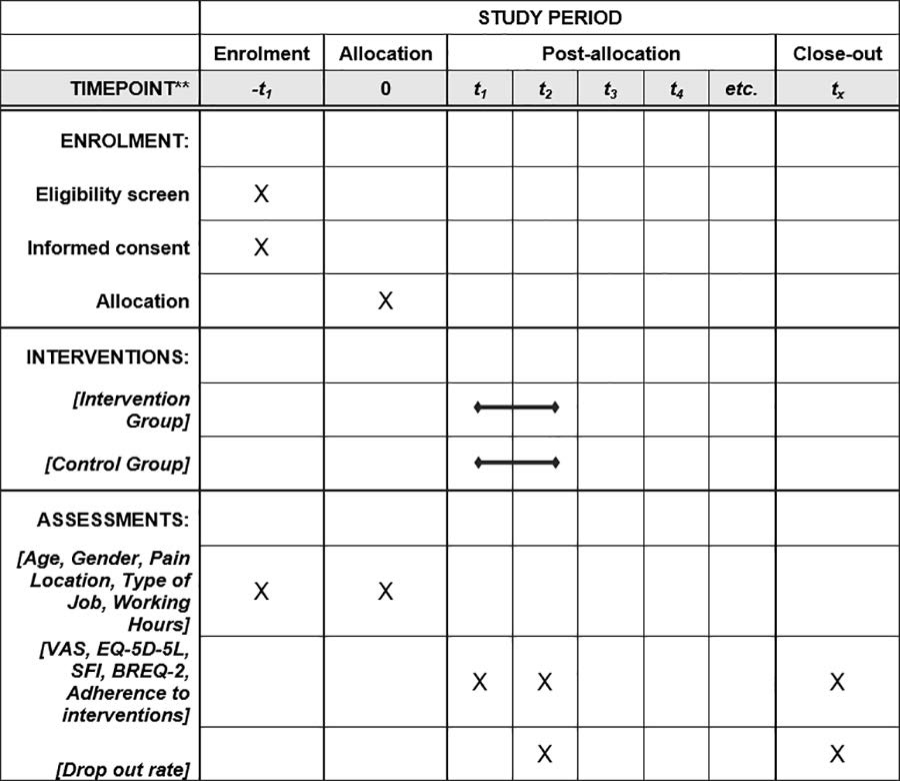
SPIRIT schedule of enrolment, interventions, and assessments. Schedule of enrolment, allocation, interventions, and data collection based on the SPIRIT 2013 guidelines for the present study.

The mixed methods study description was developed following the guidelines of the Good Reporting of a Mixed Methods Study (GRAMMS) statement [64], and the SPIRIT Checklist and Schedule (Fig 1) for reporting clinical trials [65].

## Discussion

The digitalisation of our society has accelerated exponentially due to the COVID-19 pandemic [66], driven by restrictions on movement, physical inactivity and sedentary lifestyles, which had already alarming levels before the pandemic occurred, and great efforts are needed to try to curb this situation [67]. An active lifestyle provides physical, cognitive and psychological benefits for people’s health [68,69]. Workers with long working hours and low physical activity levels in their tasks have lower daily activity levels than the average population [70], so taking advantage of breaks at work by taking active breaks can be an interesting option.

The possibilities offered by digitalisation, such as internet access and applications that remind workers via email or push notifications to take breaks, can facilitate the implementation of active breaks [71]. Research has shown that digital health interventions provide widely accessible, cost-effective, and scalable methods for managing musculoskeletal conditions, significantly reducing pain and improving functional disability [72]. In particular, tools such as audio-visual resources, emails or video calls allow patients to follow treatment plans remotely, providing flexibility and convenience [73]. While these interventions can be a significant step forward by helping patients take control of their recovery, self-manage their conditions, and maintain adherence to treatment, the broader applicability of these digital self-management tools remains uncertain, especially given the limited evidence on cost-effectiveness and patient diversity [74].

Studies have highlighted the importance of multi-component interventions that consider individual and contextual factors in reducing sitting time for long-term behavioural change [75,76]. In addition, influencing motivational aspects of sitting time reduction, both automatic and reflective motivation, is key to engaging workers in this type of intervention [77]. Considering these insights, the proposed workplace exercise intervention tends to incorporate automatic motivation through reminders and reflective motivation through educational components to help workers understand the importance of reducing musculoskeletal pain. This approach is supported by research suggesting that both types of motivation can significantly enhance engagement and adherence to interventions, particularly those focused on increasing physical activity [78]. This dual approach aligns with SDT [28], which posits that autonomous motivation (e.g., internalised health goals) enhances long-term engagement, while controlled motivation (e.g., external prompts) facilitates initial behaviour change.

This framework is supported by digital health research, highlighting the effectiveness of combining intrinsic and extrinsic motivational strategies. Peters et al [79], emphasise that digital health interventions designed with SDT principles, including autonomy-supportive elements, personalised feedback, and gamification, improve engagement and adherence. Similarly, other studies have found that integrating reflective processes (e.g., self-monitoring and goal-setting) with automatic cues (e.g., digital nudges and notifications) significantly enhances sustained behaviour change [80,81].

There are no clear guidelines on the optimal dose of physical activity for chronic pain treatment, but reducing sedentary time and increasing movement are known to be beneficial [82]. There is uncertainty about which exercise interventions in treating chronic pain might be the best option, but what has been observed is that a biopsychosocial approach is effective [83]. However, despite being low-cost interventions that are feasible to implement in the workplace, some factors can act as barriers, such as workload or organisational aspects [84]. A preliminary phase using semi-structured interviews to determine the expectations and beliefs of office workers will help us ensure that the subsequent intervention with physical exercise is adapted to the needs of this group and the context in which they find themselves during their working day.

Adopting a sequential exploratory mixed-methods approach allows for a more comprehensive understanding of workplace interventions by integrating qualitative insights with quantitative evaluation. Unlike the workplace interventions included in the current systematic review on this topic [17], which primarily implement predefined exercise programs without detailing how they were selected or tailored to workers’ needs, this study actively integrates workers’ experiences through a qualitative phase [85]. This ensures that the intervention aligns with their real-world constraints and expectations, enhancing feasibility and adherence. Mixed-methods studies are gaining relevance in the field of public health [86], with some studies suggesting that a multidimensional approach needs to be considered in the current framework to achieve relevant results [86,87]. There are other mixed methods protocols in musculoskeletal pathology in chronic low back pain [88] and women with fibromyalgia [89] that focus on this approach in which a qualitative intervention is carried out to understand the expectations and context of the patients, before designing the quantitative intervention subsequently.

### Limitations

Patients who volunteer for a research study tend to be more motivated, a common bias in studies where an informed consent form is required. This fact may affect the study’s internal validity since workplace interventions and factors such as stress, workload, or dysfunction may affect the performance of this type of intervention under normal conditions.

The results will be limited to workers who spend more than 6 hours sitting during their working day at the University of Lleida; external validity may be affected depending on the characteristics of work and the context of other sedentary workers. In addition, the use of a convenience sampling method, based on participants’ availability during working hours, may further limit generalisability. However, this approach was necessary to ensure the feasibility of recruitment within the occupational setting.

This study does not include specific tools to clinically classify pain mechanisms. However, as the target population consists of active workers with chronic non-specific spinal pain (not clinical patients on medical leave) high levels of pain severity or disability are not expected. The focus remains on functional improvement in a real-world occupational context.

## Conclusions

The study’s findings are expected to hold significant promise, particularly in understanding the importance of exercise adherence in office workers with spinal pain. By carefully considering participants’ viewpoints, the study aims to address the challenges associated with performing active pauses, especially in the dynamic workplace environment, with all its demands and stressors. The findings have the potential to shed light on how implementing such interventions can alleviate chronic pain and enhance the overall quality of life for workers, thus making a meaningful contribution to promoting health and well-being in the workplace.

## Supporting information

S1 File Semi-structured interview script. Guide used to conduct interviews with administrative staff reporting back discomfort, including open-ended questions exploring workplace experiences, physical activity, and perceived barriers.
(PDF)

S2 File Informed Consent Document. Document provided to participants detailing the purpose, procedures, risks, and rights associated with the study, as part of the informed consent process.
(PDF)

S1 Checklist Recommended items to address in a clinical trial protocol and related documents*. (DOCX)
