# Supplementary material for: A web-based workplace exercise intervention among office workers with spinal pain: Protocol of a mixed methods study
Source: PLoS One. 2025 Jun 9;20(6):e0325376. doi: 10.1371/journal.pone.0325376 (PMC12148155; doi:10.1371/journal.pone.0325376)
Supplement: S2 File — Document provided to participants detailing the purpose, procedures, risks, and rights associated with the study, as part of the informed consent process. (PDF) [file Pone.0325376.s002.pdf]

Título del proyecto: Beneficios en la reducción del dolor de espalda en personal administrativo sedentario mediante la implementación de ejercicio y educación terapéutica en el puesto de trabajo: Estudio de metodología mixta secuencial.

Investigador Principal: Carlos Tersa Miralles.

Investigadores: Cristina Bravo Navarro, Maria Masbernat Almenara, Filip Bellon, Francesc Rubí Carnacea, Esther Rubinat Arnaldo.

DOCUMENTO DE INFORMACIÓN PARA EL PARTICIPANTE

EN QUÉ CONSISTE:

Los trabajadores de oficina tienen predisposición a padecer molestias de espalda debido a ser una actividad sedentaria y al mantenimiento de la misma postura durante mucho rato seguido. Otros factores relacionados en el trabajo como la carga laboral, el clima y las expectativas en relación a la ergonomía del puesto de trabajo pueden afectar negativamente en la salud del trabajador. La fisioterapia nos ayuda a disminuir estas molestias y dolores mediante la utilización de herramientas como son el ejercicio y la educación terapéutica, en la literatura encontramos diversos estudios que implementan pausas activas en este grupo de trabajadores mostrando beneficios tanto a nivel de disminución de dolor como mejoras en la funcionalidad y la calidad de vida. Gracias a la digitalización y las facilidades que nos brinda el uso de smartphones y ordenadores, se pueden realizar intervenciones en el puesto de trabajo sin la necesidad de que el fisioterapeuta este de forma presencial mediante recursos audio-visuales. Sin embargo, uno de los problemas que nos enfrentamos como fisioterapeutas es la falta de adherencia a las intervenciones en ejercicio terapéutico, no solo por parte de los trabajadores de oficina o personas con hábitos sedentarios, sino por parte de la población en general.

Con el presente estudio queremos averiguar si el uso de ejercicio y educación terapéutica teniendo en cuenta las expectativas de los trabajadores es beneficioso en la reducción del dolor de espalda y la mejora de la funcionalidad y calidad de vida, además de aumentar la adherencia a la realización de las pausas activas siendo una práctica motivadora para ellos/ellas.

CÓMO SE REALIZA:

En una primera fase se procederá a la realización de entrevistas semi-estructuradas con el objetivo de conocer las expectativas del personal administrativo con molestias de espalda en cuanto a su dolor, la postura en el trabajo y en la aplicación de ejercicio en el horario laboral. Se realizará el registro de la imagen y la voz, mediante cámara de video y grabación de audio, para posteriormente transcribirlo en formato documento de texto.

En la segunda fase, los participantes que formen parte del grupo de intervención realizarán un tratamiento de ejercicio de 6 semanas, en el que se implementarán pausas activas junto con píldoras informativas. La dinámica de las pausas activas se implementará según los datos obtenidos en la primera fase del estudio en la que se tendrán en cuenta los ritmos de trabajo/descanso y el

tiempo que podrían dedicar a las pausas activas durante la jornada laboral. Las pausas activas consistirán en ejercicios de fuerza y movilidad de intensidad baja-moderada sin tener que desplazarse del puesto de trabajo, con el uso del propio cuerpo y en ocasiones utilizando material como la silla o la mesa para apoyar las manos o alguna otra parte del cuerpo. Los trabajadores tendrán que cumplimentar una serie de cuestionarios y escalas al inicio y al final de la intervención sobre el dolor, funcionalidad de espalda, calidad de vida y motivación al realizar ejercicio terapéutico. Por lo que hace a la adherencia, los trabajadores tendrán que llevar un control mediante un diario apuntando si han hecho las pausas o no a lo largo de los descansos propuestos.

Los participantes que se encuentren en el grupo control, se les pedirá que sigan con su actividad laboral y del tiempo libre sin modificaciones durante el transcurso de las 6 semanas que dure la intervención. Una vez acabado con el estudio, se les ofrecerá la posibilidad de realizar la misma intervención que los participantes del grupo intervención.

QUÉ EFECTOS LE PRODUCIRÁ:

La realización de ejercicio de baja-moderada intensidad no tiene consecuencias negativas. Puede notar sensación de alivio y bienestar como efecto de la liberación de endorfinas al realizar ejercicio y por la movilización del cuerpo al romper el patrón de la postura mantenida aumentando el riego sanguíneo evitando los efectos de la inmovilización de determinadas estructuras del cuerpo.

EN QUE EL BENEFICIARÁ:

Si usted tiene molestias o dolor de espalda y acepta participar en este estudio, podría beneficiarse de una disminución del dolor y una mejora de la funcionalidad, calidad de vida y motivación a la hora de realizar actividad física en su día a día, aunque esto no le podemos garantizar.

QUÉ RIESGOS TIENE:

Aunque los ejercicios, como ya hemos mencionado, no tienen consecuencias negativas que se conozcan, sí conocemos algunos riesgos relacionados. Si realizará el entrenamiento siguiendo las pautas detalladas, se minimizan mucho estos riesgos. De todas formas, le detallamos a continuación para que los conozca.

- LOS MÁS FRECUENTES:

• Riesgo 1: Aparición de dolor muscular de aparición tardía, comúnmente conocido como “agujetas”. Puede presentarse como una sensación de dolor y rigidez en los músculos afectados, y es una respuesta normal del cuerpo a la actividad física. Este tipo de dolor muscular suele desaparecer por sí solo después de unos días y no suele ser motivo de preocupación.

• Riesgo 2: Fatiga o molestias articulares derivadas del ejercicio. La sensación de molestia es parecida a la del dolor muscular de aparición tardía y desaparece al cese de la realización del ejercicio. En el caso de persistir incluso después de las primeras sesiones, es importante reportarlo para ajustar el tipo o el volumen de ejercicios en el caso de ser necesario.

- LOS MÁS GRAVES:

• Riesgo 3: Caídas. Las consecuencias de las caídas pueden ser desde leves golpes o raspones hasta lesiones más graves, como fracturas. Por esta razón, es importante tomar medidas de seguridad al realizar los ejercicios, como tener un espacio despejado y seguro, utilizar calzado adecuado y prestar atención a las indicaciones del vídeo de ejercicios. Si se presenta alguna caída o lesión durante la realización de los ejercicios, puede ser necesario buscar atención médica para recibir tratamiento adecuado.

• Riesgo 4: Cefaleas o mareos. Pueden estar relacionadas con la tensión muscular, la fatiga o la deshidratación. También pueden deberse a una mala postura o al esfuerzo excesivo durante el ejercicio. Si el dolor de cabeza o el mareo persiste o es muy intenso, es importante reportarlo y en el caso de ser necesario ponerse en contacto con el médico de cabecera o especialista para recibir un tratamiento adecuado.

- SE APLICARÁN LAS SIGUIENTES MEDIDAS PARA CADA UNO DE LOS MISMOS:

• Riesgo 1-2: Puede sentir molestias en las primeras sesiones por la falta de costumbre en la realización de ejercicio de movilidad y de fuerza, pero el periodo de adaptación suele ser de pocos días. En el caso de que no haya una mejoría a las 72 horas de haber realizado ejercicio, se recomienda consultar con el médico o fisioterapeuta de confianza para una valoración individualizada.

• Riesgo 3: En caso de que el participante tenga la sensación de falta de equilibrio al realizar los ejercicios se le modificaran para poder realizarlos sentados o con las dos piernas apoyadas siempre en el suelo.

• Riesgo 4: Si el participante reporta sensación de cefaleas o mareos en alguno de los ejercicios es importante que lo reporte para poder modificar dicho ejercicio o sustituirlo por otro parecido.

- SITUACIONES ESPECIALES A TENER EN CUENTA:

• Cualquier sensación extraña en la realización de los ejercicios o falta de seguridad es importante reportarlo para poder solucionar el problema de la forma más individualizada posible.

RECOMENDACIONES:

- No hace falta ropa de deporte para la realización de las pausas, aunque se recomienda una ropa cómoda y calzado plano o descalzarse para realizar la actividad, ya que de esta forma trabaja la musculatura intrínseca de los pies y las piernas.

INFORMACIÓN PARA EL TRATAMIENTO DE DATOS DE CARÁCTER PERSONAL

En virtud de lo que disponen los artículos 4, 5 y 6 de la Ley Orgánica 3/2018 de 5 de diciembre, de Protección de Datos de Carácter Personal (LOPD), la Universidad de Lleida y el Institut de Recerca Biomèdica de Lleida (UdL-IRBLleida) pone en su conocimiento que el hecho de firmar el presente documento implica el conocimiento y aceptación, por su parte, que la entidad dispone de un fichero

con datos de carácter personal denominado FICHERO DE INVESTIGACIÓN. La finalidad de su creación es la de gestionar los datos necesarios para la investigación que lleva a cabo la UdL-IRBLleida mediante investigadores de la Universitat de Lleida y del Grupo de Investigación en Cuidados de la Salud (GRECS), garantizando el registro y seguimiento de la prestación asistencial que requerirán los usuarios durante el estudio y obteniendo información para cumplimentar la Historia Clínica de los usuarios. Para garantizar el anonimato, cada participante recibirá un código de participante único. No se mencionarán datos personales en ningún archivo, excepto en los formularios de consentimiento informado, que se archivarán en una carpeta separada y se guardarán en un gabinete cerrado. Solo el investigador principal podrá tener acceso a esta información, la cual podrá ser utilizada con el objetivo de ponerse en contacto con el/la paciente, en el caso de las incidencias que puedan ocurrir durante el ensayo. Los datos anónimos se pueden compartir en conferencias y publicaciones. Los participantes serán informados sobre esto a través de su formulario de consentimiento. Los destinatarios de la información son todos los departamentos en que se organiza la UdL-IRBLleida así como los estamentos oficiales públicos o privados que, por obligación legal o necesidad material, tengan que acceder a los datos a los efectos del correcto desarrollo del proyecto de investigación, de acuerdo con las buenas prácticas científicas. En todo caso, tiene derecho a ejercitar los derechos de oposición, acceso, rectificación y cancelación en el ámbito reconocido por la LOPD. El responsable del fichero es el director gerente de la entidad. Para ejercitar los derechos anteriormente mencionados, y para cualquier aclaración, puede dirigirse por escrito mediante carta dirigida al director gerente.

Si le surge cualquier duda o pregunta sobre el estudio, estamos siempre a su disposición y puede ponerse en contacto directamente con el Investigador Principal, Carlos Tersa Miralles en el teléfono 676 20 17 43; o en la dirección de correo electrónico carles.tersa@udl.cat

Los participantes pueden ejercer sus derechos en relación con los datos personales proporcionados en el presente documento contactando con el Delegado de Protección de Datos de la Universitat de Lleida a través del teléfono 973 702 197 o del correo electrónico dpd@udl.cat . Asimismo, cualquier duda o consulta en materia de protección de datos personales puede ser resuelta a través de esta vía de contacto.

Título del proyecto: Beneficios en la reducción del dolor de espalda en personal administrativo sedentario mediante la implementación de ejercicio y educación terapéutica en el puesto de trabajo: Estudio de metodología mixta secuencial.

Investigador Principal: Carlos Tersa Miralles.

Investigadores: Cristina Bravo Navarro, Maria Masbernat Almenara, Filip Bellon, Francesc Rubí Carnacea, Esther Rubinat Arnaldo.

Este documento sirve para que usted, dé su consentimiento para participar en este estudio. Esto significa que nos autoriza a realizar esta intervención. Usted puede retirar este consentimiento cuando lo desee. Firmarlo no le obliga a participar en el estudio. De su rechazo no se derivará ninguna consecuencia adversa respecto a la calidad del resto de la atención médica recibida. Antes de firmar, es importante que haya leído atentamente la información contenida en la hoja informativa del estudio, que ha recibido junto con este consentimiento. Si tiene alguna duda o necesita más información no dude en decirnos lo, le atenderemos con mucho gusto.

Consentimiento informado:

DATOS DEL PACIENTE

Apellidos y nombre del / de la paciente:

PROFESIONAL QUE INTERVIENE EN EL PROCESO DE INFORMACIÓN Y / O CONSENTIMIENTO:

Apellidos y nombre: Firma:

Fecha:

Consentimiento: Yo, D / Dña..., manifiesto que estoy conforme con el estudio que se me ha propuesto. He leído y comprendido la información contenida en la hoja informativa que se me ha proporcionado. He podido preguntar y aclarar todas mis dudas. Por eso he tomado consciente y libremente la decisión de participar. También sé que puedo retirar mi consentimiento cuando lo estime oportuno.

A el de de

El/La PACIENTE

Firmado:

Revocación del consentimiento:

Yo, ..., de forma consciente y libre he decidido retirar mi consentimiento a participar en este estudio.

A el de de

El/La PACIENTE

Firmado:
